# Mental Health of Working Adults during the COVID-19 Pandemic: Does Physical Activity Level Matter?

**DOI:** 10.3390/ijerph20042961

**Published:** 2023-02-08

**Authors:** Tai-Ming Wut, Stephanie Wing Lee, Jing (Bill) Xu

**Affiliations:** College of Professional and Continuing Education, The Hong Kong Polytechnic University, Hong Kong, China

**Keywords:** level of physical activity, perceived mental well-being, depression, anxiety, self-esteem, working adults, COVID-19 pandemic

## Abstract

The purpose of this study is to investigate the associations between physical activity levels and the psychological outcomes of depression and anxiety. In 2022, Hong Kong was still exercising strict measures to control the spread of COVID-19. In this connection, major events and almost all large-scale sports events were suspended. Most recreational facilities were closed and repurposed as vaccination venues. As a result, a reduction in physical activity was expected. A cross-sectional survey was conducted among 109 working adults in Hong Kong. The International Physical Activity Questionnaire-Short Form was adopted as it continues to be the most widely used scale to measure physical activity. Almost a quarter of respondents exercised regularly. On average, respondents engaged in less than an hour’s physical activity per week. Findings showed that even low to moderate levels of physical activity were positively associated with perceived self-esteem and perceived mental well-being. More specifically, self-esteem and perceived mental well-being were negatively associated with depression and anxiety. A full mediation effect between engagement in low levels of physical activity and anxiety was found. Light exercises may ultimately lead to lower anxiety via an indirect effect, with perceived mental well-being acting as a mediator. There was no direct relationship between low levels of physical activity and anxiety. In a similar vein, moderate levels of physical activity may lead to amelioration of symptoms related to depression and anxiety through indirect effects, with self-esteem as a mediator. Apart from engagement in low levels of physical activity, moderate levels of physical activity, such as swimming, jogging, and dancing, which have associations with self-esteem and mental health, could also be considered for attention.

## 1. Introduction

Around 25% of adults fail to engage in sufficient physical activity [1]. With the outbreak of the COVID-19 pandemic, many individuals are now working from home and staying indoors most of the time, even on weekends [2]. Ever since the first identification of COVID-19 cases in Mainland China in December 2019, Hong Kong and Macau have been high-risk regions due to geographical proximity and frequent exchange of people, as suggested by the increasing number of confirmed infection cases. Coronavirus is a highly contagious disease that can spread widely and rapidly through close interpersonal contact. To avoid the gathering of people, individuals are advised to maintain the practice of social distancing. Some of them continue to work from home. As a result, they might spend more time on the computer and mobile phone, either for work, news, or entertainment. Increased screen time and reduced physical activity have been suggested to be associated with heightened risks of poor sleep quality and mental health problems [3]. Many parts of the world, including Europe and the United States, have already reopened their borders and cancelled social distancing restrictions in 2022. Only Mainland China, Macao, and Hong Kong are still exercising strict control to halt the COVID-19 pandemic. Most of the recreational facilities were closed and repurposed as vaccination venues. As a result, a reduction in physical activity was expected [4].

### 1.1. Mental Health

“Mental health”, as defined by the World Health Organization (2004), is “a state of well-being in which the individual realizes his or her own abilities, can cope with the normal stresses of life, can work productively and fruitfully, and is able to make a contribution to his or her community” [5]. Mental health comprises emotional, psychological, and social well-being. It affects how individuals think, feel, and act. It also helps determine how people handle stress, relate to others, and make choices. Mental health is important at every stage of life, from childhood and adolescence through adulthood [6].

Engagement in physical activity has shown to improve mental well-being [7,8,9]. Individuals are more prone to mental health issues during the lockdown period. Due to the limited home living space in cities, individuals may not be able to engage in high amounts of different sports and activities, such as badminton, table tennis, squash, swimming, or even jogging. In this regard, this study set out to investigate the associations between physical activity levels and the psychological outcomes of depression and anxiety.

### 1.2. Research Framework

**Figure 1:**
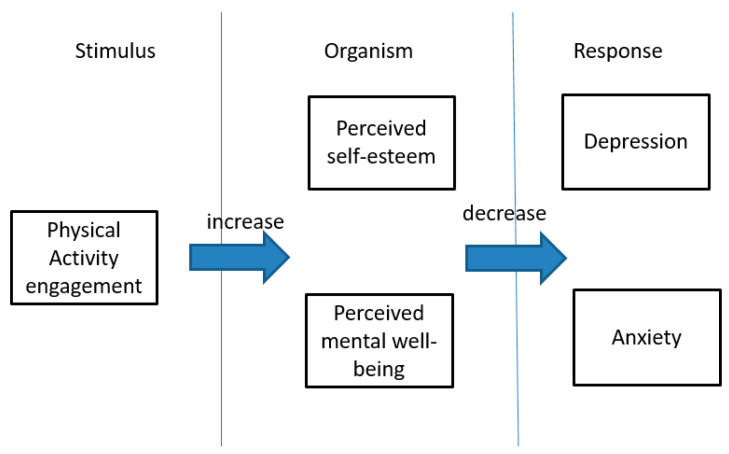
Conceptual framework proposed (source: authors).

Our proposed overarching logic of the framework was based on stimulus-organism-response (SOR) theory (Figure 1). It has been used to predict psychological behavior [2]. Physical activity is a type of stimulus. Perceived self-esteem and mental well-being are intermediate states. Depression and anxiety are outcomes or responses. Various levels of physical activity may increase people’s perceived self-esteem and mental well-being, which could in turn reduce depression and anxiety.

### 1.3. What Has Been Carried out So Far

Research studies on the relationship between physical activity and mental health have been conducted. For example, a moderately positive correlation between resilience and mental health and a weakly positive correlation between resilience and physical activity were found [8]. Individuals who engaged in physical activity had better mental health compared to those who did not [9]. Therefore, it was concluded that engagement in physical activity may be an important contributory factor towards the mental health of working adults. Mild or light exercises such as yoga, fishing, bowling, golf, archery, and easy walking are preferred by working adults as such exercises entail quick recovery [10]. Engagement in easy exercises during the weekend does not affect their work schedules or performance.

A cross-sectional study was conducted to examine the relationship between physical activity and mental well-being in undergraduate nursing students. The results showed that self-esteem was significantly positively correlated with total and moderate-intensity physical activity [11]. No other significant relationships were found among anxiety, depression, and physical activity. Outcome expectations for exercise and self-efficacy were positively correlated with moderate- and vigorous-intensity physical activity and total physical activity.

Moderate-level physical activities include fast walking, baseball, tennis, volleyball, badminton, easy swimming, skiing, and popular and folk dancing, which may require at least a day’s rest for full recovery [10]. A large-scale study investigating the relationship between physical activity and mental health in one million individuals found that more exercise does not always equate to better mental health [12]. Rather, an optimal range in terms of intensity was recommended.

Research findings have indicated that self-esteem remains one of the most important outcomes associated with subjective well-being [13]. High self-esteem has been shown to be linked with positive outcomes, including mental well-being, higher job satisfaction, and better social relationships [14]. A meta-analysis reviewing existing studies indicates that low self-esteem is a risk factor for various negative outcomes, including anxiety and depression [13]. Scholars agree that physical activity might improve people’s mental health and reduce depression [15]. Having low self-esteem makes an individual more susceptible to developing depression subsequently [16].

The prolonged implementation of COVID-19 social distancing measures brought about a mental health tsunami in Hong Kong, in which individuals’ mental health deteriorated to the lowest level in 8 years. About 41% of survey respondents expressed that their mental health was negatively affected in a similar manner to the 2019 social unrest in Hong Kong [17]. In response to the social movement, the percentage of respondents reporting post-traumatic stress disorder (PTSD) rose from 5% in 2015 to almost 32% in 2019 [18]. Similar to the global population, around 25% of adults in Hong Kong do not engage in sufficient physical activity. In a population-based survey conducted by the Department of Health between November 2020 and November 2022, involving more than 16,000 adults in 7400 domestic households, compared to previous survey findings, a significant increase in physical inactivity among adults was observed, which may be attributed to the COVID-19 pandemic [19]. Moreover, due to the socioeconomic downturn, further fueled by high interest rates and harsh restriction measures, people felt increasingly insecure in terms of safety and livelihood [2]. The effect of physical activity is also applicable to people with severe mental illness [20]. However, the mechanism underlying the effect of physical activity on anxiety is yet to be uncovered [21]. Our paper thus sheds light on the research gap by investigating the associations between physical activity level and the psychological outcomes of depression and anxiety.

### 1.4. Development of Hypotheses

**Figure 2:**
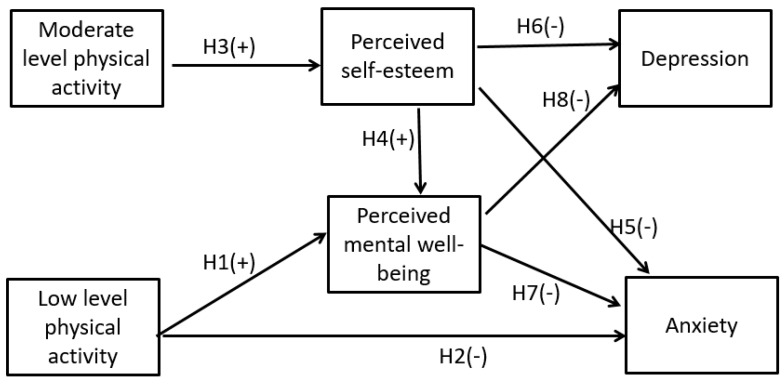
Research model (source: authors). Note: H indicates hypothesis; + sign indicates positive association; and - sign indicates negative association.

The full research model (Figure 2) is presented below, along with the proposed hypotheses. Various levels of physical activity were proposed to be associated with perceived mental well-being and self-esteem. The psychological outcomes of depression and anxiety were hypothesized to be negatively associated with perceived mental well-being and self-esteem.

**Hypothesis 1.**  *Low level of physical activity is positively associated with perceived mental well-being*.

**Hypothesis 2.**  *Low level of physical activity is negatively associated with anxiety*.

**Hypothesis 3.**  *Moderate level of physical activity is positively associated with perceived self-esteem*.

**Hypothesis 4.**  *Self-esteem is positively associated with mental well-being*.

**Hypothesis 5.**  *Self-esteem is negatively associated with anxiety*.

**Hypothesis 6.**  *Self-esteem is negatively associated with depression*.

**Hypothesis 7.**  *Perceived mental well-being is negatively associated with anxiety*.

**Hypothesis 8.**  *Perceived mental well-being is negatively associated with depression*.

## 2. Materials and Methods

A cross-sectional survey was conducted from August to September 2022 among working adults in Hong Kong. Due to feasibility concerns, the questionnaire was distributed using online means. Before the main survey, a pilot study was conducted in June 2022. Some adjustments had been made to the wording of items in the questionnaire. For the main survey, two reminders were sent to maximize the number of responses and thus increase the response rate. All the data collected were automatically recorded by the system and manually transcribed and coded in an Excel spreadsheet for analysis.

### 2.1. Measurement

Measurement items from five established, self-reported scales on amount of physical activity performed, self-esteem, depression, anxiety, and mental well-being were used, respectively.

#### 2.1.1. Physical Activity

The International Physical Activity Questionnaire-Short Form was chosen [22] as it is the most widely used scale for measuring physical activity. It possesses high validity and reliability in assessing the frequency, intensity, and duration of self-reported physical activity in the past week [10,23]. To facilitate the input of answers by respondents, the questions on durations for all categories of physical activities were changed from a fill-in-the-blank format to a multiple-choice format. In particular, the response options for daily sitting time aligned with those adopted in the survey of sitting time conducted by [24]. Some examples of physical activities were replaced by exercises that are more conveniently and frequently performed by Hong Kong people [25].

#### 2.1.2. Self-Esteem

Self-esteem was measured by the Rosenberg Self-Esteem Scale (RSE) [26], a 10-item instrument scored on a 4-point scale from strongly agree to strongly disagree, which was expanded to a 5-point scale to maintain the consistency of this study. This is a well-known, reliable, and validated tool for measuring global self-esteem [27], which is widely used in all populations [28]. In this study, participants were asked to answer questions focusing on the past week (7 days).

#### 2.1.3. Depression

The Center for Epidemiologic Studies Depression Scale (CES-D) [29] consists of 20 items rated on a scale from 0 to 3. Respondents are asked to rate how often they experienced symptoms associated with depression over the past week. In this study, the original 4-point scale was converted to a 5-point scale (1 to 5), with 1 = “rarely or none of the time (less than 1 day)” to 5 = “Most or all of the time (7 days)”, the exact number of days was provided for respondents’ convenience and was calculated on a pro rata basis. The CES-D has also been shown to possess satisfactory psychometric properties as an instrument assessing depressive symptoms in Chinese participants [30,31].

#### 2.1.4. Anxiety

Anxiety was measured using the Generalized Anxiety Disorder 7-item scale (GAD) [32], which is a self-administered 7-item questionnaire measuring the severity of various signs of generalized anxiety disorder for screening purposes. A recent study conducted in the Korean undergraduate population showed that the GAD-7 possesses high internal consistency and good convergent validity [33]. In the original GAD, respondents were instructed to report on their experience during the past two weeks, whereas in this study, participants were asked to answer questions in relation to the past week (7 days). The original 4-point scale was converted to a 5-point scale to maintain consistency with other scales in this study. The wording of the response options was aligned with those suggested by [34].

#### 2.1.5. Perceived Mental Well-Being

The short version of the Warwick–Edinburgh Mental Well-being Scale (SWEMWBS) [35] was employed for its conciseness and reliability. The SWEMWBS contains 7 statements selected from the 14 items in the long version of the WEMWBS with 5 response options, from 1 = “none of the time” to 5 = “all of the time”. Adequate internal consistency and reliability were demonstrated in a study examining its use in Norway and Sweden [36].

### 2.2. Data Collection

Convenience sampling was used. Data were collected from working adults belonging to various industries using a Google Form between August and September 2022 (Table A1). Before data collection, ethical approval was obtained from the College of Professional and Continuing Education Research Committee. Respondents were told the purpose of the study and invited to participate in a voluntary manner. Respondents could withdraw from the study at any time. Contact information was provided in case of further queries. A total of 200 electronic mails were sent out, and 109 valid responses were collected. The response rate was 54.5%. An incentive equivalent to USD 6.00 was given to participants who successfully completed the survey.

### 2.3. Data Analysis

Partial least squares structural equation modeling (PLS-SEM) was used for data analysis. The PLS-SEM method explains the variance of dependent variables, which fits the purpose of this study. This method is suitable for non-normal data and small sample sizes. With a minimum path coefficient of 0.21 to 0.30 and a 5% significance level, the recommended minimum sample size is 69. This method entails two steps, with the first step testing the reliability and validity of the measurement model and the second step testing the structural model [37]. The sample size of our study thus fulfilled the analysis requirements.

### 2.4. Demographic Information of Respondents

Around 60% of respondents were female and 40% were male. A total of 45.9% of respondents were aged between 18 and 30 years old, while 29.4% of respondents were aged between 41 and 50 years old. Around one-third of respondents were professionals, including doctors, accountants, and auditors. Most of the doctor respondents were male, while most of the accountant and auditor respondents were female. The remaining respondents worked in finance, logistics, information technology, engineering, marketing, non-governmental organizations, and government sectors. A total of 43.1% of the respondents were in entry-level positions; 17.4% were at supervisory levels; and 18.3% were at middle-management levels (Table 1). According to the latest census statistics for 2021, 44% of the Hong Kong population is male and 56% is female. In terms of occupation, almost 30% are professionals (our sample: 36%), 11.8% are from manufacturing and construction industries (our sample: 11%), and 3.4% are from information technology (our sample: 5.5%). Our percentages are lower for trading and logistics, social services, and financial services. The average age of the population is 46, while the average age of our sample is 41 [38].

It was found that 24 respondents (22.01%) did not have any regular exercise habits. On average, respondents engaged in less than an hour of physical activity per week (Table 2).

## 3. Results

Partial least squares structural equation modeling consists of two stages, with the first stage testing the reliability and validity of the measurement model and the second stage testing the structural model [37].

### 3.1. Reflective Measurement Model

The reflective measurement model assessment is shown below in Table 3.

Outer indicator loadings lower than the threshold of 0.708 were dropped [39]. Two indicator loadings of the anxiety construct, eight indicator loadings of the depression construct, and one indicator loading of the self-esteem construct were thus deleted. All of Cronbach’s alphas were higher than 0.7. In particular, the Cronbach’s alpha for the depression construct was 0.953, which was close to the maximum. The average variance of the extracted values was all greater than 0.5, indicating good convergent validity. The discriminant validity assessment using the Fornell–Larcker criterion is shown in Table 4 below. The values in diagonal cells were greater than the remaining values, indicating good discriminant validity.

### 3.2. Structural Model Assessment

**Figure 3:**
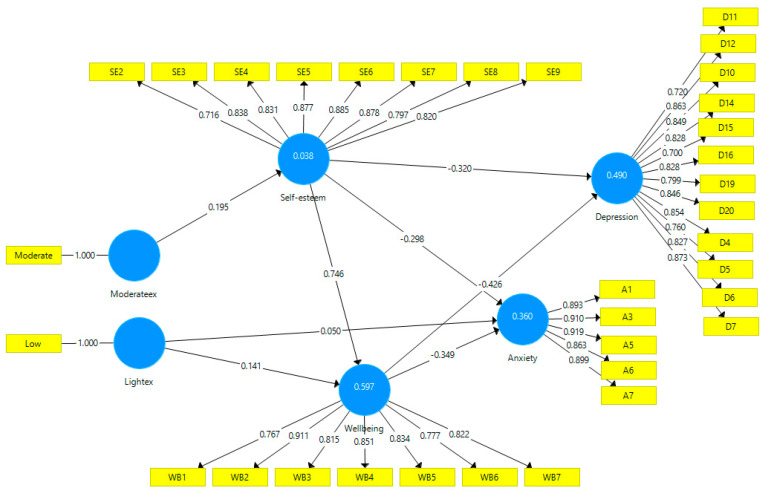
PLS structural model (source: authors).

A partial least squares model is shown in Figure 3 below. All the paths were found to be significant. All collinearity values were less than 5, indicating the absence of collinearity problems [39]. The adjusted R-squared values for depression, anxiety, and mental well-being were 0.481, 0.342, and 0.591, respectively, indicating that 34.2% to 59.1% of the variance was explained. The R-squared value of self-esteem was 0.038, indicating that 3.8% of the variance was explained.

## 4. Hypothesis Testing

Anxiety and depression were the outcome variables of our research model. Mental well-being and self-esteem were the intermediate variables. Hypothesis 1 proposed an association between light exercise and mental well-being. As shown in Table 5, the hypothesis was supported. Hypothesis 2 proposed a negative association between light exercise and anxiety. However, this hypothesis was not supported. Hypothesis 3 proposed an association between moderate exercise and self-esteem, which was supported as expected. Hypotheses 4, 5, and 6 proposed associations between self-esteem and mental well-being, anxiety, and depression, respectively. These three hypotheses were all supported. Finally, Hypotheses 7 and 8 proposed associations between mental well-being and anxiety and depression, respectively. These two hypotheses were supported.

## 5. Discussion

This study investigated the associations among levels of physical activity and the psychological outcomes of depression and anxiety. All the hypotheses were supported, except Hypothesis 2. Hypothesis 1 proposed a positive relationship between low levels of physical activity and mental well-being. As expected, they had a positive association. Engagement in light exercises such as fishing, yoga, and easy walking enhances mental well-being. This result is consistent with the existing literature findings [9].

Hypothesis 2 proposed a negative association between engagement in light exercise and anxiety. No significant relationship between engagement in light exercise and anxiety was found. It is possible that when working adults are engaged in easy walking and yoga, they may still be mentally preoccupied with daily hassles. Since such physical activities are not of vigorous intensity, individuals can still spare some mental capacity to pay divided attention to other matters, e.g., to scroll on their mobile phones or check electronic mail. The results concur with previous findings that people with severe mental illness tended to engage in less vigorous exercise [20].

Hypothesis 3 proposed a positive relationship between moderate levels of physical activity and self-esteem. As expected, they were positively associated, thus concurring with previous findings [11]. Engagement in moderate exercises such as fast walking, baseball, tennis, volleyball, badminton, and low-intensity swimming enhanced self-esteem. However, the variance explained was low, suggesting that there may be other factors affecting self-esteem. Possible factors may be personality and expectations of reward and punishment [40].

Hypothesis 4 suggested a positive association between self-esteem and mental well-being. As expected, they were associated with each other, thus concurring with previous findings [13,14]. Hypothesis 5 suggested a negative association between self-esteem and anxiety. Our findings supported the hypothesis and were similar to those of [13,41].

Hypothesis 6 proposed a negative association between self-esteem and depression, which was supported by our findings. Our results aligned with those of [42,43]. Hypotheses 7 and 8 proposed negative associations between mental well-being, and anxiety and depression, respectively. As expected, the hypotheses were supported by our findings.

**Figure 4:**
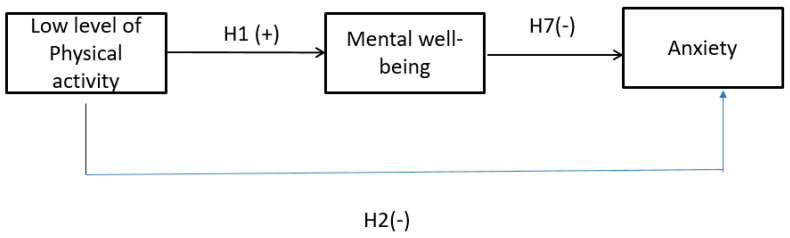
Full mediation effect. Note: H means hypothesis; + sign means positive association; and - sign means negative association.

A full mediation effect between engagement in low levels of physical activity and anxiety was found (Figure 4). There was a significant relationship between engagement in light exercise and perceived mental well-being (Hypothesis 1). Perceived mental well-being was also negatively associated with anxiety (Hypothesis 7). The relationship between engagement in light exercise and anxiety was found to be non-significant (Hypothesis 2). Thus, a full mediation effect was established. In other words, better perceived mental well-being would help suppress anxiety, which is logical as people could know how to handle stress and remain calm in crisis [2].

### 5.1. Theoretical Contribution

Several theoretical contributions are proposed. First, this study provides an explanation of how engaging in light and moderate exercise leads to various psychological outcomes, such as amelioration of symptoms related to depression and anxiety. Our results show that there is no direct relationship between light exercise and anxiety. However, performing low levels of physical activity may ultimately lead to lower anxiety via an indirect effect, with perceived mental well-being acting as a mediator. In a similar vein, moderate levels of physical activity may lead to amelioration of symptoms related to depression and anxiety through indirect effects, with self-esteem as a mediator. A more complicated route is that a moderate level of physical activity leads to lower anxiety through a chain of mediators involving self-esteem and mental well-being. Finally, empirical support demonstrates that higher levels of physical activity are associated with more health benefits, concurring with previous findings [8,9].

### 5.2. Managerial Implication

Companies are recommended to organize low-level physical activities such as yoga or Pilates courses for employees to reduce work stress. Such activities could even be organized during work hours in order to encourage participation. Furthermore, apart from engagement in low-intensity physical activities, moderate-intensity physical activities such as swimming, jogging, and dancing, which have associations with self-esteem and mental health, could also be considered.

## 6. Conclusions

This study investigated the associations between physical activity levels and the psychological outcomes of depression and anxiety. Low levels of exercise were positively associated with perceived mental well-being, while moderate levels of physical activity were positively associated with perceived self-esteem. Perceived mental well-being and self-esteem were negatively associated with depression and anxiety, respectively.

There are several limitations pertaining to our study. First, researchers adopted a cross-sectional study design, and thus causality cannot be established. Second, there may be other factors affecting perceived mental well-being and self-esteem that were not investigated in this study. Third, self-reported data were used. Finally, control variables such as industry types, tenure, and levels of employees were not included. The above-mentioned variables could be added to our future research models as extensions for further research. Further research could investigate how different types of sports exert differential psychological effects on employees from various job sectors. The duration of physical activity could be further explored as well. A large-scale longitudinal study would also be useful in establishing causality.

## Figures and Tables

**Table 1:** Demographic data of respondents.

Category		Frequency	Percentage %
Gender	Male	43	39.4
	Female	66	60.6
Age	18–30	50	45.9
	31–40	14	12.8
	41–50	32	29.4
	51–60	10	9.2
	61 or above	3	2.8
Industry	Tourism	5	4.6
	Financial services	2	1.8
	Trading and logistics	7	6.4
	Construction	2	1.8
	Information technologies	6	5.5
	Engineering	10	9.2
	Retail or marketing	3	2.8
	Customer services	5	4.6
	Professional services	36	33
	Cultural and creative industries	2	1.8
	Civil servant	4	3.7
	Non-government organization	4	3.7
	Others	23	21.1
Job level	Entry-level	47	43.1
	Supervisory level	19	17.4
	Middle-management level	20	18.3
	Senior-management level	10	9.2
	Director level	1	0.9
	Self-employed	12	11

**Table 2:** Descriptive statistics of physical activity levels among working adults.

Physical Activity Intensity (Mins/Week)	Light Intensity PA	Moderate Intensity PA	Vigorous Intensity PA	Total PA
Self-reported physical activity—Mean	29.4	17.3	10	56.3
Standard deviation	30.0	23.52	19.07	--

**Table 3:** Measurement model assessment.

Construct	Item	Loading	Cronbach’s Alpha	Composite Reliability	AVE
Anxiety	A1	0.893	0.939	0.954	0.804
	A3	0.910			
	A5	0.919			
	A6	0.862			
	A7	0.899			
Depression	D4	0.853	0.953	0.959	0.663
	D5	0.743			
	D6	0.827			
	D7	0.854			
	D10	0.836			
	D11	0.710			
	D12	0.863			
	D14	0.817			
	D15	0.711			
	D16	0.833			
	D19	0.804			
	D20	0.841			
Self-esteem	SE2	0.736	0.936	0.947	0.692
	SE3	0.838			
	SE4	0.823			
	SE5	0.875			
	SE6	0.875			
	SE7	0.874			
	SE8	0.797			
	SE9	0.806			
Perceived mental well-being	WB1	0.767	0.922	0.938	0.683
	WB2	0.911			
	WB3	0.815			
	WB4	0.852			
	WB5	0.835			
	WB6	0.777			
	WB7	0.822			

**Table 4:** Assessing discriminant validity (Fornell–Larcker criterion).

Constructs	Mean	Standard Deviation	1	2	3	4
1. Anxiety	2.117	1.033	0.897			
2. Depression	2.128	0.850	0.881	0.814		
3. Self-esteem	3.633	0.778	−0.558	−0.643	0.832	
4. Mental well-being	3.491	0.827	−0.565	−0.669	0.760	0.827

**Table 5:** Results of hypotheses testing.

Hypothesis	Item	(*β*) Path Coefficient	*t*-Value	*p*-Value	Result
H1	Light exercises >> Perceived mental wellbeing	0.141	2.812	0.005 **	Supported
H2	Light exercises >> Anxiety	0.050	0.547	0.584	Unsupported
H3	Moderate exercises >> Self-esteem	0.195	1.991	0.047 *	Supported
H4	Self-esteem >> Perceived mental well-being	0.746	13.309	0.000 ***	Supported
H5	Self-esteem >> Anxiety	−0.298	2.048	0.041 *	Supported
H6	Self-esteem >> Depression	−0.320	2.764	0.006 *	Supported
H7	Perceived mental well-being >> Anxiety	−0.349	2.210	0.027 *	Supported
H8	Perceived mental well-being >> Depression	−0.426	3.392	0.001 **	Supported

(Bootstrap samples = 5000, *n* = 109 cases). * *p* < 0.05; ** *p* < 0.01; and *** *p* < 0.001.

## Data Availability

Data are available upon request.

## Appendix A

**Table A1:** Modified survey items.

Construct Name and Source	Items
Depression [29]	I was bothered by things that usually do not bother me. I did not feel like eating; my appetite was poor. I felt that I could not shake off the blues even with help from my family friends. I felt I was just as good as other people. I had trouble keeping my mind on what I was doing. I felt depressed. I felt that everything I did was an effort. I felt hopeful about the future. I thought my life had been a failure. I felt fearful. My sleep was restless. I was happy. I talked less than usual. I felt lonely. People were unfriendly. I enjoyed life. I had crying spells. I felt sad. I felt that people dislike me. I could not get “going”.
Anxiety [32]	Feeling nervous, anxious or an edge Not being able to stop or control worrying Worrying too much about different things Trouble relaxing Being so restless that it is hard to sit still Becoming easily annoyed or irritable Feeling afraid as if something awful might happen
Self-esteem [26]	On the whole, I am satisfied with myself. At times I think I am no good at all. I feel that I have a number of good qualities. I am able to do things as well as most other people. I feel I do not have much to be proud of. I certainly feel useless at times. I feel that I am a person of worth, at least on an equal plane with others. I wish I could have more respect for myself. All in all, I am inclined to feel that I am a failure.
Perceived mental well-being [35]	I have been feeling optimistic about the future I have been feeling useful I have been feeling relaxed I have been dealing with problems well I have been thinking clearly I have been feeling close to other people I have been able to make up my own mind about things
